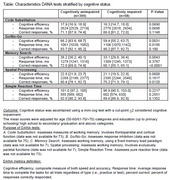# Digital version of Defense Automated Neurobehavioral Assessment in South Asian Setting: Preliminary evidence from the P‐CARRS study

**DOI:** 10.1002/alz.087168

**Published:** 2025-01-09

**Authors:** Ram Jagannathan, Rani Komal, Deepa Mohan, Poongothai Subramani, Rima Pai, Suvarna Alladi, Sailesh Mohan, Mohammed K Ali, Nikhil Tandon, Dorairaj Prabhakaran, V Mohan, Allan I. Levey, Venkat Narayan KM, Felicia Goldstein

**Affiliations:** ^1^ Hubert Department of Global Health, Rollins School of Public Health, Atlanta, GA USA; ^2^ Centre for Chronic Disease Control, New Delhi, Delhi India; ^3^ Madras Diabetes Research Foundation, Chennai, Tamil Nadu India; ^4^ Hubert Department of Global Health, Rollins School of Public Healt, ATLANTA, GA USA; ^5^ National Institute of Mental Health and Neuro Sciences, Bengaluru, Karnataka India; ^6^ All India Institute of Medical Sciences, New Delhi, Delhi India; ^7^ Dr. Mohan's Diabetes Specialities Centre and Madras Diabetes Research Foundation, Chennai India; ^8^ Emory University School of Medicine, Atlanta, GA USA; ^9^ Emory University, Atlanta, GA USA

## Abstract

**Background:**

The Defense Automated Neurobehavioral Assessment (DANA) encompasses a suite of standardized neurocognitive screening tools designed for detecting various neurodegenerative diseases and subtle cognitive deficits. This study presents a pilot investigation into digital cognitive screening, utilizing an Android version of the DANA tests, conducted among a diverse South Asian population residing in India.

**Methods:**

The study involved individuals aged over 50 years, nested within the ongoing population‐based longitudinal Precision‐CARRS study, representative of socio‐demographically and linguistically diverse adults from Delhi and Chennai in India. Cognitive functioning was assessed using the Android‐based mobile platform DANA screening library: code substitution (set shifting), Go/No‐Go (response inhibition), memory search (working memory), spatial processing (visuospatial), and simple reaction time (processing speed), facilitated by a commercially available FDA‐approved Linus Health digital application. To classify participants as cognitively impaired or intact, performance on the traditionally administered paper/pencil version of the Mini‐Cog screening test, which includes clock drawing and three‐item recall, was considered.

**Results:**

A total of 358 participants (age, median: 61.0 years; range: 50‐89 years; female: 56%; educational categories: up to primary schooling: 8.4%; high school to secondary: 65.9%; graduation and above: 25.7%) with the Mini‐Cog screening test and smartphone‐based DANA screening were included in the analysis. Sixteen percent (n=58) of the participants were cognitively impaired on the Mini‐Cog (0‐2 points/5 total points). On average, DANA tasks were administered in ∼20 minutes. Older age (p<0.0001) was associated with worse DANA cognitive efficiency scores, response time, and fewer cumulative correct responses. There were no sex differences in performance, and only two of the five DANA task performances (code substitution and memory search) differed based on education categories. Compared to those without impaired Mini‐Cog scores, the impaired group had decreased cognitive efficiency scores, slower average response times, and fewer correct responses for two (Go/No‐Go and memory search) of the five DANA tasks (Table).

**Conclusion:**

Our pilot study strongly demonstrates the potential of using smartphone‐based standardized DANA assessments in cognitive screening and the scalability of digital assessments in a diverse population from a resource‐constrained LMIC setting.